# Associations of Subjective Age Trajectories With Loneliness and Stress Across Adulthood

**DOI:** 10.1093/geroni/igaf122.223

**Published:** 2025-12-31

**Authors:** Anna Kornadt, Markus Wettstein, Anthony Lepinteur, Claus Vögele, Conchita D’Ambrosio

**Affiliations:** University of Luxembourg, Esch-sur-alzette, Grevenmacher, Luxembourg; Humboldt-Universität zu Berlin, Berlin, Berlin, Germany; University of Luxembourg, Esch-sur-alzette, Grevenmacher, Luxembourg; University of Luxembourg, Esch-sur-alzette, Grevenmacher, Luxembourg

## Abstract

Subjective age, that is the age a person feels compared to their chronological age, is indicative of a variety of aging processes. Studies that investigate multi-variate, dynamic, longitudinal relations of subjective age with potential determinants and mechanisms of these relations have rarely been employed. In the current study, we focus on loneliness as a potential subjective age determinant, as loneliness affects a variety of psychosocial and health outcomes across life and is stereotypically perceived as a feature of old age. We investigate whether loneliness is related with levels and changes in subjective age and test whether this association is mediated via self-reported stress. N = 5,594 adults aged 18 – 93 years (Mage = 50.41, SD = 15.99) who participated in a longitudinal survey comprising up to three measurement occasions over a time span of 2.5 years reported their loneliness, subjective age, and stress as well as sociodemographic and health-related covariates. We employed latent growth modeling and found that, when controlling for sociodemographic and health-related covariates, higher loneliness was related to an older subjective age cross-sectionally and a to steeper increase in subjective age over time. These relations were mediated via stress; however, the relation between stress and subjective age was no longer statistically significant when including the covariates. All associations were qualified by significant interactions with chronological age, albeit in different directions. Our findings attest to the associations between loneliness, stress and subjective aging experiences and highlight the need for an age-informed approach when planning further studies and interventions.

